# Dentoskeletal effects of clear aligner vs twin block—a short-term study of functional appliances

**DOI:** 10.1007/s00056-022-00443-1

**Published:** 2023-01-18

**Authors:** Elisabetta Cretella Lombardo, Roberta Lione, Lorenzo Franchi, Francesca Gaffuri, Cinzia Maspero, Paola Cozza, Chiara Pavoni

**Affiliations:** 1 Department of Systems Medicine, University of Rome ‘Tor Vergata’, Viale Oxford 81, 00133 Rome, Italy; 2 Department of Dentistry, UNSBC, Tirana, Albania; 3 Department of Surgery and Translational Medicine, University of Florence, Florence, Italy; 4 Department of Biomedical, Surgical and Dental Sciences, University of Milan, Fondazione IRCCS Ca’ Granda Ospedale Maggiore Policlinco, Milan, Italy; 5 UniCamillus–Saint Camillus International University of Health Sciences, Rome, Italy

**Keywords:** Class II malocclusion, Orthodontic appliances, removable, Mandibular advancement, Mandibular skeletal retrusion, Twin block, Klasse-II-Malokklusion, Herausnehmbare kieferorthopädische Apparaturen, Vorverlagerung des Unterkiefers, Retrusion des Unterkieferskeletts, Twin-Block

## Abstract

**Purpose:**

The twin block (TB) is one of the most widely used functional appliances for the correction of class II malocclusions. Align Technology (San Jose, CA, USA) developed the Invisalign® mandibular advancement (MA) that replicates the mechanism of action of a functional appliance. The aim of this study was to compare the changes produced by the TB versus those by MA.

**Methods:**

The records of 56 class II patients treated with the TB (TB group: *n* = 35) or the MA (MA group: *n* = 21) were compared to a control sample of 15 untreated class II subjects (UC2).

**Results:**

The TB and MA groups showed a significant reduction of the ANB angle, compared to the controls (TB group: −1.5°; MA group: −1.5°; UC2 group: +0.2°). For the Co-Gn values, the TB and MA groups showed significant differences when compared with the UC2 group with an increase of 8.4 mm in TB patients and of 8.3 mm in MA patients. The increase of the distance of Pg to the true vertical line (TVL) was the only measurement where significant differences between the three groups were found with a greater advancement of the soft tissue pogonion in the TB group compared with the MA group and the UC2 group (TB group: +3 mm; MA group: +0.9 mm; UC2 group: −1.6 mm). The angle between the palatal plane and mandibular plane revealed a more relevant reduction in the TB and MA groups. Both appliances were able to reduce overjet and vertical overbite values.

**Conclusions:**

Treatment with the MA and TB appliances produced a significant elongation of the mandible with an improvement in sagittal relationship, overjet, and vertical overbite and with good control of the vertical relationship. TB subjects showed a greater advancement of the soft tissue chin.

## Abstract (de)

**Zielsetzung:**

Der Twin-Block (TB) ist eine der am häufigsten verwendeten funktionellen Apparaturen für die Korrektur von Klasse-II-Fehlstellungen. Align Technology (San Jose, CA, USA) hat das Invisalign® Mandibular Advancement (MA) entwickelt, die den Wirkmechanismus einer funktionellen Apparatur nachahmt. Ziel dieser Studie war es, die Veränderungen durch die TB mit denen durch die MA zu vergleichen.

**Methoden:**

Die Datensätze von 56 mit TB (TB-Gruppe: *n* = 35) bzw. MA (MA-Gruppe: *n* = 21) behandelten Klasse-II-Patienten wurden mit denen einer Kontrollgruppe (UC2) von 15 unbehandelten Klasse-II-Patienten verglichen.

**Ergebnisse:**

Die TB- und MA-Gruppen zeigten eine signifikante Verringerung des ANB-Winkels im Vergleich zur Kontrollgruppe (TB-Gruppe: -1,5°; MA-Gruppe: -1,5°; UC2-Gruppe: +0,2°). Bei den Co-Gn-Werten zeigten die TB- und MA-Gruppen signifikante Unterschiede im Vergleich zur UC2-Gruppe mit einer Zunahme von 8,4 mm bei den TB-Patienten und von 8,3 mm bei den MA-Patienten. Die Zunahme des Abstands von Pg zur „true vertical line“ (TVL) war der einzige Messwert, bei dem ein signifikanter Unterschied zwischen den 3 Gruppen festgestellt wurde, mit einer größeren Vorverlagerung des Weichgewebepogonions in der TB-Gruppe im Vergleich zur MA-Gruppe und der UC2-Gruppe (TB-Gruppe: +3 mm; MA-Gruppe: +0,9 mm; UC2-Gruppe: -1,6 mm). Der Winkel zwischen Gaumenebene und Unterkieferebene zeigte eine stärkere Verringerung in der TB- und der MA-Gruppe. Beide Apparaturen waren in der Lage, den Overjet und den vertikalen Overbite zu reduzieren.

**Schlussfolgerungen:**

Die Behandlung mit den MA- und TB-Apparaturen führte zu einer signifikanten Verlängerung des Unterkiefers mit einer Verbesserung von Sagittalstellung, Overjet und vertikalem Overbite und mit einer guten Kontrolle der vertikalen Beziehung. Bei den TB-Patienten wurde eine stärkere Vorverlagerung des mentalen Weichgewebes festgestellt.

## Introduction

Class II malocclusion can be a result of maxillary protrusion, mandibular retrusion, or a combination of factors [1–3]. When class II, division 1 malocclusion is associated with mandibular skeletal retrusion, a viable treatment option is the alteration of the amount and direction of mandibular growth by using functional appliances. Several functional appliances have been specifically designed to enhance mandibular growth and forward repositioning of the mandible in order to correct class II dentoskeletal disharmonies [4–8].

One of the most often applied functional device for the resolution of class II skeletal malocclusions is the twin block (TB) appliance developed by Clark. The TB consists of two removable plates overlapping with each other with inclined acrylic surfaces that lead the lower jaw forward during bite closure [9].

The two-phase treatment of class II skeletal malocclusions has been considered as a suitable treatment approach in growing patients [10, 11]; this therapy comprises growth modification with functional therapy followed by orthodontic treatment with fixed appliances.

However, in the last few years, clear aligner therapy continued to increase its scope from the simplest cases to malocclusions requiring orthognathic surgery, dental extractions, and nowadays also functional appliances for growing patients with class II malocclusion [12–14]. Align Technology (San Jose, CA, USA) developed a functional appliance using their patented materials that combines the concepts of growth modification with active tooth movement in the anterior region, becoming one of the two treatment phases.

Released in late 2017, Invisalign® mandibular advancement (MA) reproduced the mechanisms of action of functional appliances as it leads the mandible forward. The advancement movement occurs through an engagement of inclined planes built into buccal precision wings placed between the first molars and premolars when the patient occludes. Align Technology conceived the MA for growing patients with mild to severe retrognathic class II malocclusion and with a permanent dentition or a stable late mixed dentition.

In the literature, only one study [15] directly compared the effects resulting from MA and TB treatments; however, a limitation was the lack of untreated controls. Thus, this retrospective study aimed to compare the dentoskeletal changes resulting from treatment using the MA and the TB compared with an untreated control group with the same type of malocclusion.

## Methods

The study project was approved by the Ethics Committee at the University of Tor Vergata, and informed consent was obtained from the subjects’ parents for the treatment and for the potential use of their data for research purposes. Sample size was calculated for the analysis of variance (ANOVA) test considering to detect a difference in the primary outcome variable ANB of 1.7° with a standard deviation of 1.4° [16], an alpha (α) value of 0.05 and a power of 0.80. At least 15 subjects were required for each group (SigmaStat 4.0, Systat Software Inc., San José, CA, USA).

In this retrospective, controlled clinical trial, the cephalometric records of 56 patients with class II division 1 malocclusion treated consecutively either with the TB (TB group: *n* = 35, 17 males, 18 females; mean age 12.0 ± 1.3 years), or the MA (MA group: *n* = 21, 9 males, 12 females; mean age 11.2 ± 1.1 years) were collected. Class II subjects were retrieved from the records of patients treated at the Department of Orthodontics at the University of Tor Vergata (TB) and at the University of Milan (MA).

The subjects were selected according to the following criteria: overjet between 5 mm and 8 mm, bilateral full class II or end-to-end molar relationships, ANB angle greater than 4°, improvement in facial profile when the lower jaw was postured in a forward position, cervical stage 3 in cervical vertebral maturation (CVM) at T1. Lateral cephalograms were available at two time periods: T1, at the start of treatment; and T2 at the end of functional therapy, before orthodontic therapy with both fixed appliance or the continuing phase with additional aligners. Functional treatment was discontinued after the achievement of class I molar relationship.

A total of 15 subjects with untreated class II division 1 malocclusion were selected from the American Association of Orthodontists Foundation Craniofacial Growth Legacy Collection (http://www.aaoflegacycollection.org, Bolton–Brush Growth Study, Michigan Growth Study, Denver Growth Study, Oregon Growth Study, and Iowa Growth Study; UC2 group: *n* = 15, 4 males, 11 females; mean age 10.9 ± 1.1 years).

The treated and the control samples were selected according to skeletal maturity at the start of treatment as evaluated by means of the CVM. The CVM method can be used to identify individual skeletal maturity in growing subjects, and it can replace the hand–wrist radiograph. CVM staging was performed by an expert examiner (CP) [17]. The three groups were matched in terms of age and gender. The demographic data of the MA group, TB group, and UC2 group are reported in Table 1.

**Table 1:** Demographics for the treatment and control groups Demografische Charakteristika der Behandlungs- und Kontrollgruppen

	T1	T2
	Mean age ± SD	Mean age ± SD
Group TB (*n* = 35, 17F, 18M)	12.0 ± 1.3	13.8 ± 1.3
Group MA (*n* = 21, 12F, 9M)	11.2 ± 1.1	13.7 ± 1.3
Group UC2 (*n* = 15, 11F, 4M)	10.9 ± 1.1	13.0 ± 0.7

*TB* twin block, *MA* mandibular advancement, *UC2* untreated class II subjects, *SD* standard deviation, *F* female, *M* male

All patients were treated by two expert clinicians. The clinical experience of the two operators in the management of the two functional appliances was similar on the basis of years of experience and number of patients treated with functional appliances.

### Treatment protocol

**Fig. 1:**
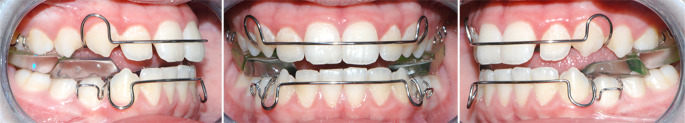
Frontal and lateral views of a twin block (TB) appliance Vorder- und Seitenansicht einer Twin-Block-Apparatur (TB)

Patients of the TB group were treated with a TB device constructed following the design originally conceived by Clark (Fig. 1; [18]). The appliance was comprised of maxillary and mandibular plates that fit against the teeth, alveolus, and other supporting structures. Delta or Adams clasps were constructed on both sides to anchor the upper plate to the first permanent molars, and 0.030-inch ball clasps (or arrow clasps) were positioned in the interproximal spaces anteriorly. The precise clasp arrangement depended on the state of the dentition at the moment of TB construction. In the mandibular arch, Clark suggested placing ball hooks in the interproximal areas between the canines and incisors [18].

For all patients beginning TB therapy, the devices were realized from bite registrations taken with the incisors in an end-to-end position when the starting overjet was within 7–8 mm. If the initial overjet was greater than 8 mm, a two-step activation was carried out with the initial bite registration taken halfway between centric relation and incisal end-to-end position, with subsequent activation to edge-to-edge relationship 3–4 months later. Essentially, the construction bite was obtained to permit 5–7 mm of vertical opening in the area of the posterior bite blocks. An important advantage of the twin block is the opportunity to guide vertical eruption of posterior teeth through selective removal of acrylic throughout the therapy. In hypodivergent subjects with a short lower anterior facial height and/or a deep curve of Spee, the acrylic on the posterior area of the upper bite block was trimmed to encourage the eruption of the lower posterior teeth. All subjects included in the present research were recommended to wear the device full time for a minimum of 22 h a day (with the exception of meals and sports) until the end of therapy. The second phase of treatment consisted of full-fixed appliance therapy in the permanent dentition.

**Fig. 2:**
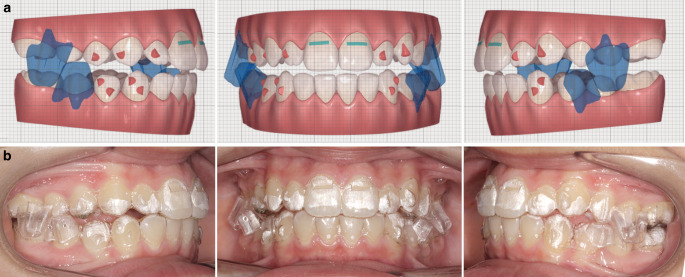
Frontal and lateral views of a mandibular advancement (MA) appliance: **a** Clin-check plan and **b** intraoral view Frontale und laterale Ansichten einer Unterkiefervorschubapparatur (MA): **a** Clin-Check-Plan und **b** intraorale Ansicht

Patients of the MA group were treated with the Mandibular Advancement (MA) appliance (Fig. 2). The aligners feature precision wings made from the patented SmartTrack® material that are situated between the premolars and first molars. The wings engage and hold the mandible in a forward position. The jaw shifts incrementally forward into its proper place. While the aligners are at work on the orthopedic correction, they also simultaneously align and level the teeth.

An initial pre-MA phase was done automatically in specific situations (deep bite > 7 mm, molar rotation > 20°, class II division 2, and cross-bite) to allow wing placement or to allow the first advancement to take place [19].

After mandibular advancement the transitional phase was planned to hold the mandible in the advanced position while waiting for standard aligners or additional aligners to be delivered.

The standard treatment phase represented the last phase of treatment which include additional standard aligners to finalize the occlusion: finish leveling the curve of Spee, correct any remaining dental class II malocclusion and coordinate and detail the arches.

As with regular aligners treatment, patients were instructed to wear the aligners a minimum of 22 h a day, only being removed to eat, drink, brush, and floss. Aligners were changed weekly.

### Cephalometric analysis

**Fig. 3:**
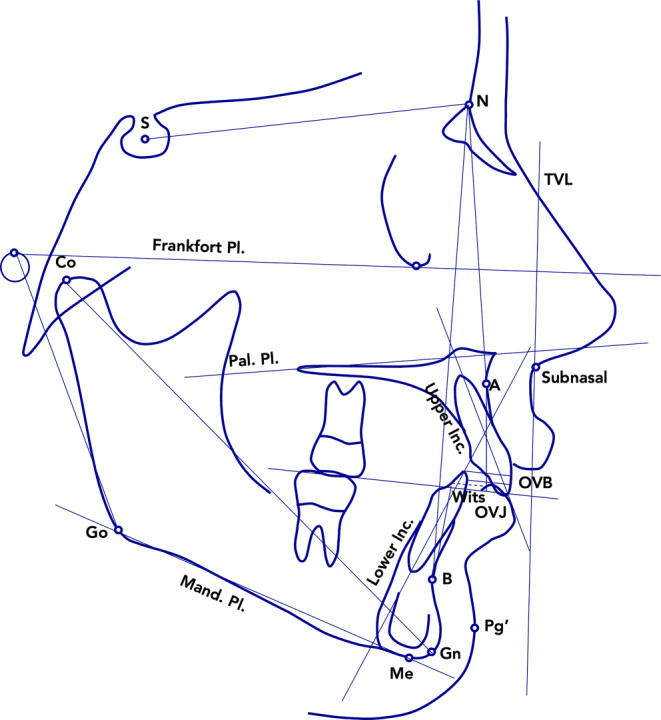
Cephalometric parameters measured at T1 and at T2. *S* sella, *N* nasion, *Pal* palatal, *Pl.* plane, *Mand.* mandibular, *Inc.* incisor, *OVJ* overjet, *OVB* vertical overlap, *TVL* true vertical line (perpendicular to the Frankfort Plane and passing through point subnasal), *Go* gonion, *Co* condilion, *Me* menton, *Gn* gnation, *Pg’* soft tissue pogonion, *A* point A, *B* point B Zu den Zeitpunkten T1 und T2 gemessene kephalometrische Parameter. *S* Sella, *N* Nasion, *Pal* palatinal, *Pl.* Ebene, *Mand.* mandibular, *Inc.* Incisivus, *OVJ* Overjet, *OVB* senkrechter Overlap, *TVL* „true vertical line“ (senkrecht zur Frankfurter Horizontale und durch den Punkt Subnasale), *Go* Gonion, *Co* Condilion, *Me* Menton, *Gn* Gnathion, *Pg’* Weichgewebe-Pogonion, *A* Punkt A, *B* Punkt B

Lateral cephalograms were hand traced at a single sitting by one investigator (ECL). This investigator calibrated for landmark definition by a second investigator (CP) before digitization. A customized digitization regimen (Viewbox, version 4.0, dHAL Software, Kifissia, Greece) was created and used for cephalometric evaluation. Lateral cephalograms for each patient at T1 and T2 were digitized, and a custom cephalometric analysis was used. Fourteen variables (5 linear and 9 angular) were generated for each tracing (Fig. 3).

### Statistical analysis

Fisher exact test was used to assess differences in gender distribution between the three groups. Descriptive statistics and statistical comparisons between the TB group, the MA group, and the UC2 group at T1 (starting forms) and for the T2–T1 changes were assessed by means of the analysis of variance (ANOVA) with Tukey’s post hoc test. When the variables were not normally distributed (Shapiro–Wilk test), Kruskal–Wallis with Dunn’s post hoc tests were performed [20].

### Method error

Fifteen lateral cephalograms, selected randomly, were remeasured after a washout period of 2 weeks by the same operator (ECL). Intraobserver reproducibility was assessed with the intraclass correlation coefficient (ICC), while for the assessment of the random error the method of moments’ estimator (MME) was applied [21].

## Results

The values for the ICCs varied from 0.720–0.993, indicating substantial to almost perfect intrarater agreement [21]. The MME random error measurements ranged from 0.3 to 1.0° for the angular variables and from 0.3 to 0.8 mm for the linear measurements. Gender distribution in the three groups was not statistically different (Fisher exact probability test *p* = 0.397).

As reported in Table 2, the analysis of the staring forms at T1 showed statistically significant differences between the three groups only for overjet and vertical overbite values. No significant differences were found at T1 for the other linear and angular measurements.

**Table 2:** Descriptive statistics and statistical comparisons of baseline characteristics. Analysis of variance (ANOVA) with Tukey’s post hoc tests or ANOVA on ranks with Dunn’s post hoc tests Deskriptive Statistiken und statistische Vergleiche der Ausgangsbedingungen. Varianzanalyse (ANOVA) mit Tukeys Post-Hoc-Tests bzw. Rang-ANOVA mit Dunns Post-Hoc-Tests

Variables	TB group (1) (*n* = 35)		MA group (2) (*n* = 21)		Control group (3) (*n* = 15)		*P*	Multiple test comparisons								
	Mean	SD	Mean	SD	Mean	SD		1 vs 2			1 vs 3			2 vs 3		
*Sagittal skeletal*								Diff	*P*	95% CI	Diff	*P*	95% CI	Diff	*P*	95% CI
SNA (°)	81.1	3.2	82.2	2.6	81.7	2.7	0.415	−1.1	0.389	−3.0 to 0.8	−0.6	0.804	−2.8 to 1.6	0.5	0.870	−1.9 to 2.9
SNB (°)	74.7	3.4	76.0	2.0	75.5	2.5	0.238	−1.3	0.221	−3.2 to 0.6	−0.8	0.658	−2.9 to 1.3	0.5	0.836	−1.8 to 2.9
ANB (°)	6.4	1.7	5.4	1.4	6.0	1.4	0.076	1.0	0.061	0.0 to 2.1	0.4	0.658	−2.9 to 1.3	−0.6	0.520	−1.9 to 0.7
Wits (mm)	3.0	2.1	2.7	1.8	2.7	2.7	0.849	0.3	0.879	−1.1 to 1.7	0.3	0.895	−1.3 to 1.9	0.0	1.000	−1.7 to 1.8
Co-Gn (mm)	100.3	5.7	101.9	6.5	98.7	5.7	0.286	−1.6	0.614	−5.5 to 2.4	1.6	0.644	−2.7 to 6.0	3.2	0.257	−1.6 to 8.0
TVL-Pg’ (mm)	−10.9	4.1	−8.9	4.6	−8.7	2.7	0.095	−2.0	0.181	−4.6 to 0.7	−2.2	0.170	−5.2 to 0.7	−0.2	0.977	−3.5 to 3.0
*Vertical skeletal*																
SN-Pal. Pl. (°)	8.6	2.5	7.4	2.6	7.2	3.7	0.149	1.2	0.266	−0.6 to 3.1	1.4	0.227	−0.6 to 3.5	0.2	0.970	−2.0 to 2.5
SN-Mand. Pl. (°)	34.1	5.8	32.0	4.0	32.1	4.7	0.255	2.1	0.312	−1.3 to 5.4	2.0	0.433	−1.8 to 5.7	−0.1	0.998	−4.2 to 4.0
Pal. Pl.-Mand. Pl. (°)	25.5	6.6	22.9	5.1	25.4	5.3	0.256	2.6	0.256	−1.3 to 6.5	0.1	0.997	−1.3 to 6.5	−2.5	0.436	−7.3 to 2.3
CoGoMe (°)	124.3	6.4	121.3	4.7	121.6	4.5	0.106	3.0	0.138	−0.7 to 6.6	2.7	0.275	−1.5 to 6.8	−0.3	0.985	−4.8 to 4.2
*Dentoalveolar*																
Overjet (mm)	7.2	1.8	5.2	2.1	6.1	2.3	*0.002*	*2.0*	*0.003*		1.1	0.089		−0.9	1.00	
Overbite (mm)	4.6	1.6	4.7	1.6	2.9	1.1	*0.001*	−0.1	0.983	−1.1 to 0.9	*1.7*	*0.002*	0.6 to 2.9	*1.8*	*0.003*	0.5 to 3.0
Upper Inc.-Pal. Pl. (°)	112.4	7.6	112.0	9.6	112.2	6.1	0.980	0.4	0.977	−5.0 to 5.7	0.2	0.997	−5.7 to 6.1	−0.2	0.994	−6.7 to 6.2
Lower Inc.-Mand. Pl. (°)	97.2	5.9	100.2	9.0	97.1	7.3	0.270	−3.0	0.280	−7.8 to 1.7	0.1	1.000	−5.3 to 5.4	3.1	0.420	−2.8 to 8.9

Italic values 0.6 to 2.9

*SD* standard deviation, *Diff.* difference, *TVL* true vertical line, *Pal. Pl.* palatal plane, *Mand. Pl.* mandibular plane, *95% CI* 95% confidence interval. *TB* twin block, *MA* mandibular advancement

Descriptive statistics and statistical comparisons of the T2–T1 changes are reported in Table 3.

**Table 3:** Descriptive statistics and statistical comparisons of the T2–T1 changes. Analysis of variance (ANOVA) with Tukey’s post hoc tests or ANOVA on ranks with Dunn’s post hoc tests Deskriptive Statistiken und statistische Vergleiche der T2-T1-Veränderungen. Varianzanalyse (ANOVA) mit Tukey Post-Hoc-Tests bzw. Rang-ANOVA mit Dunnʼs Post-Hoc-Tests

Variables	TB group (1) (*n* = 35)		MA group (2) (*n* = 21)		Control group (3) (*n* = 15)		*P*	Multiple test comparisons								
	Mean	SD	Mean	SD	Mean	SD		1 vs 2			1 vs 3			2 vs 3		
*Sagittal skeletal*								Diff	*P*	95% CI	Diff	*P*	95% CI	Diff	*P*	95% CI
SNA (°)	0.1	1.7	−0.4	2.6	−0.7	1.5	0.390	0.5	0.679	−0.8 to 1.8	0.8	0.392	−0.7 to 2.2	0.3	0.865	−1.2 to 1.9
SNB (°)	1.6	1.4	1.4	3.2	0.4	1.1	0.165	0.2	0.918	1.1 to 1.6	1.2	0.145	−0.3 to 2.7	1.0	0.337	−0.7 to 2.6
ANB (°)	−1.5	1.4	−1.5	1.5	0.2	0.3	*0.000*	0.0	0.981	−0.9 to 0.8	*−1.7*	*0.000*	−2.6 to −0.7	*−1.7*	*0.001*	−2.6 to −0.6
Wits (mm)	−1.1	2.9	−0.8	2.4	0.4	2.3	0.163	−0.3	0.904	−2.0 to 1.4	−1.5	0.141	−3.5 to 0.4	−1.2	0.346	−3.3 to 0.9
Co-Gn (mm)	8.4	2.0	8.3	3.1	3.3	1.2	*0.000*	0.1	1.000		*5.1*	*0.000*		*5.0*	*0.000*	
TVL-Pg’ (mm)	3.0	2.0	0.9	3.7	−1.6	3.3	*0.000*	*2.1*	*0.027*	0.2 to 4.0	*4.6*	*0.000*	2.5 to 6.7	*2.5*	*0.033*	0.2 to 4.8
*Vertical skeletal*																
SN-Pal. Pl. (°)	−0.4	1.9	−0.3	3.6	0.2	1.2	0.866	−0.2			−0.1			0.1		
SN-Mand. Pl. (°)	−0.8	1.7	−1.7	2.0	0.9	1.1	0.663	−0.1	0.964	−1.8 to 1.4	−0.6	0.637	−2.5 to 1.1	−0.5	0.811	−2.5 to 1.5
Pal. Pl.-Mand. Pl. (°)	−0.4	2.5	−0.5	3.1	0.6	0.8	*0.000*	0.9	0.140	−0.2 to 2.0	**−***1.7*	*0.005*	−2.9 to −0.4	**−***2.6*	*0.000*	−3.9 to −1.2
CoGoMe (°)	−3.7	1.9	−2.6	2.4	0.0	0.9	0.389	0.1	0.982	−1.5 to 1.7	−1.0	0.438	−2.8 to 0.9	−1.1	0.416	−3.0 to 0.9
*Dentoalveolar*																
Overjet (mm)	−1.6	4.8	−0.5	7.0	0.0	0.0	*0.000*	−1.1	0.100	−2.3 to 0.2	**−***3.7*	*0.000*	−5.0 to −2.2	**−***2.6*	*0.000*	−4.1 to −1.0
Overbite (mm)	1.1	4.3	−1.0	7.9	0.7	1.6	*0.000*	−0.1	0.968	−1.1 to 0.9	**−***2.0*	*0.000*	−3.1 to −0.9	**−***1.9*	*0.001*	−3.1 to −0.7
Upper Inc.-Pal. Pl. (°)	0.1	1.7	−0.4	2.6	−0.7	1.5	0.079	−1.1			−1.6			−0.5		
Lower Inc.-Mand. Pl. (°)	1.6	1.4	1.4	3.2	0.4	1.1	0.345	2.1	0.323	−1.4 to 5.6	0.4	0.969	−3.5 to 4.3	−1.7	0.603	−6.0 to 2.6

Italic values great significance of the results

*SD* standard deviation, *Diff.* difference, *TVL* true vertical line, *Pal. Pl.* palatal plane, *Mand. Pl.* mandibular plane, *95% CI* 95% confidence interval, *TB* twin block, *MA* mandibular advancement

Both the TB group and MA group showed a statistically significant and clinically relevant reduction of the ANB angle, compared to the control group (TB group: −1.5° ± 1.4°; MA group: −1.5° ± 1.5°; UC2 group: +0.2° ± 0.3°).

Also for the parameter Co-Gn, the TB group and MA group showed statistically significant differences when compared with the UC2 group with a total increase of 8.4 mm in patients treated with the TB appliance and of 8.3 mm in MA patients.

The increase of the distance of Pg to TVL from T1 to T2 was the only statistically significant difference observed between the three groups with a significantly greater advancement of the soft tissue pogonion in the TB group compared with the MA group and the UC2 group (TB group: +3 mm ± 2 mm; MA group: +0.9 mm ± 3.7 mm; UC2 group: −1.6 mm ± 3.3 mm).

In the vertical plane, the angle between the palatal plane and mandibular plane revealed a more relevant reduction in the TB and MA groups. These results proved to be statistically significant compared with the control group.

Both functional appliances were able to reduce the overjet and the vertical overbite values from T1 to T2, with statistically significant differences with respect to the control group.

## Discussion

Traditionally, functional appliances have been used for many years in the treatment of class II malocclusion with the aim of obtaining a skeletal correction of mandibular retrusion [6–8]. Several functional appliances exist for the treatment of class II malocclusions and among these, one of the most common is the TB [9]. In recent years Align technology extended its area of expertise including functional appliances for growing patients with class II malocclusions. In 2017, Invisalign® released the MA which replicates the mechanism of action of traditional functional appliances.

To our knowledge, up to now, only one study [15] evaluated the effects of the MA with respect to the the TB. However, this study did not use a control group. Therefore, the present retrospective study aimed to compare the dentoskeletal changes obtained by using the MA and the TB with respect to untreated controls with the same type of malocclusion. Both TB and MA are based on the same mechanism of action with inclined planes that induce the mandible to assume a forced anterior position, with subsequent neuromuscular adaptation. In the present study, the decrease of the ANB angle suggests the efficacy of both devices when compared with an untreated class II control group.

In contrast with our results, the study published by Caruso et al. in 2021 reported statistically significant differences between the TB group and the MA group for the ANB angle [15]. The authors found significant differences for the short-term treatment changes (T1–T2) for each group, but the TB group showed a more relevant reduction in the ANB angle (−5.6°) when compared with MA subjects (−3.4°).

From our results, the only statistically significant difference between the TB, MA, and UC2 groups was related to the advancement of the chin evaluated by the distance of the soft tissue pogonion from the true vertical line (TVL). In the present study, the TB appliance seemed to be more efficient in the advancement of the chin and consequently in the improvement of the facial profile. This could be related to the one step protrusion of the mandible performed if using the TB compared to the gradual advancement realized with the MA.

An interesting observation reported in the study conducted by Caruso et al. is related to the position of the maxilla, showing that in MA patients there was no change of the SNA angle value which was different from the TB subjects that showed a significant reduction of this parameter [15]. This could explain the higher reduction of the ANB angle found in the cited study in the TB patients when compared with the MA subjects. In the current investigation, no statistically significant effects were reported for the changes of the maxillary position for either the TB or MA groups.

A further difference between the present study and the one conducted by Caruso et al. is related to overjet, vertical overbite, and dental inclination values. The results of the current investigation showed no differences for the changes of dental parameters between the MA and TB groups but both groups were significantly different if compared with the controls. Conversely, in the study published by Caruso et al. statistically significant differences were found between the two treatment groups for upper incisor’s inclination and consequently for overjet reduction [15]. These findings suggest that the TB appliance induced a reduction in upper incisor inclination with a consequent dentoalveolar compensation of the class II malocclusion. On the contrary, in the present sample, both appliances seemed to ensure good control of dental inclination of the incisors allowing the mandible to be advanced.

According to Align Technology, the MA appliance is indicated in growing patients with mild-to-severe retrognathic class II malocclusions who present in the permanent dentition or in a stable late mixed dentition where it is anticipated that the primary second molars will persist for the duration of the advancement phase.

Various advantages are offered by the application of the MA compared to traditional functional appliances, such as ease of use and improved esthetics, comfort, and hygiene [22]. Moreover, with the MA, dental alignment can be reached during mandibular advancement allowing an improvement of facial as well as of dental esthetics in the course of the first phase of treatment.

One major contraindication is if there are any supernumerary teeth present buccal to the premolars or permanent first molars. Due to the position of the wings on the buccal surface of the aligners and they might potentially place unwanted pressure in the region of the supernumerary teeth. As with traditional functional appliances, the main challenge is related to the compliance of the patient.

The results of the present study showed that both the TB and MA appliances are efficient in the management of class II malocclusion with a more relevant improvement of the profile induced by the TB.

A limitation of the present study is related to its short-term nature and to the small number of patients involved. However, after the recent application of this new technique (MA), further investigations are necessary to increase the sample size and to evaluate the stability of the results in the long term.

## Conclusions

Treatment of class II malocclusion with mandibular advancement (MA) and twin block (TB) functional appliances produced in the short-term period the following:

- Significant elongation of the mandible in the treatment groups compared with controls. This effect was associated with an improvement in the skeletal sagittal intermaxillary relationship and in overjet.
- Significant reduction of the vertical overbite which was associated with control of the vertical skeletal relationship in both treated groups.
- Significantly greater advancement of the chin in the TB patients associated with an improvement of the facial profile.

## Abbreviations

CVM: Cervical vertebral maturation
ICC: Intraclass correlation coefficient
MA: Mandibular advancement
MME: Moments’ estimator
TB: Twin block
UC2: Untreated class II
